# Research progress on the role of microbiome-immune-neurotransmitter network in post-stroke sleep disorders

**DOI:** 10.3389/fnagi.2025.1694709

**Published:** 2025-12-01

**Authors:** Wanting Shi, Li Wu, Qiong Qin, Yanjing Li, Wei Chen

**Affiliations:** 1 Department of Neurosurgery, Affiliated Hospital of Zunyi Medical University, Zunyi, Guizhou, China; 2 Department of Nursing, Affiliated Hospital of Zunyi Medical University, Zunyi, Guizhou, China; 3 School of Nursing, Zunyi Medical University, Zunyi, Guizhou, China

**Keywords:** stroke, sleep disorders, microbiome-immune-neurotransmitter network, gut microbiota, brain-gut axis

## Abstract

Post-stroke sleep disorders, as a significant complication affecting patient rehabilitation, are closely associated with dysregulation of the microbiome-immune-neurotransmitter network. Following stroke, activation of the hypothalamic-pituitary-adrenal axis and sympathetic nervous system triggers intestinal barrier disruption (reduced tight junction proteins and intestinal permeability) along with microbial imbalance (decreased *Bifidobacterium* and increased Enterobacteriaceae). Reduced short-chain fatty acids and lipopolysaccharide (LPS) translocation exacerbate systemic inflammatory responses and neurotransmitter imbalances (inhibited serotonin synthesis and excitotoxic glutamate production). These changes further disrupt circadian regulation by the hypothalamic suprachiasmatic nucleus, leading to reduced REM sleep and disrupted slow-wave sleep architecture. Future research should prioritize interventional strategies targeting the gut microbiota, such as probiotics, prebiotics, and fecal microbiota transplantation, integrated with multi-omics technologies and neural circuit modulation approaches, to elucidate the spatiotemporal dynamics of the microbiome–immune–neurotransmitter network and provide a theoretical basis for clinical translation. Restoring brain–gut axis homeostasis is expected to improve post-stroke sleep disorders and neurological functional outcomes in patients.

## Graphical Abstract

**Figure:**
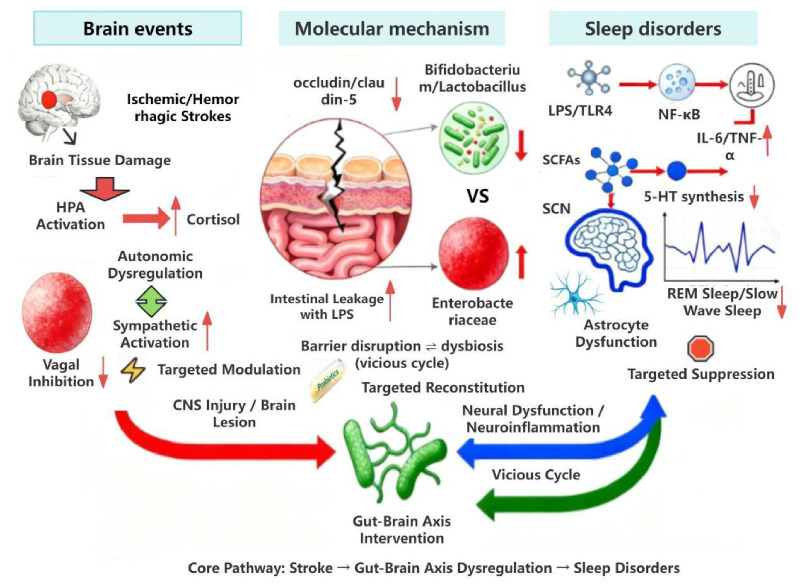
This schematic summarizes the mechanistic link between stroke and subsequent sleep disorders via gut-brain axis dysregulation. Following a stroke, brain damage induces HPA axis activation and autonomic dysfunction, which in turn disrupts the intestinal barrier and microbiota balance. The resulting gut leakage permits LPS translocation, triggering systemic inflammation through the TLR4/NF-kB pathway. This inflammation ultimately causes astrocyte dysfunction and sleep disturbances. The graphic also highlights potential intervention points, such as microbiota reconstitution and anti-inflammatory strategies.

## 1 Introduction

Stroke, an acute cerebrovascular condition encompassing ischemic and hemorrhagic types, results in brain tissue damage and exhibits high incidence, disability, and recurrence. It ranks as the second leading cause of death globally and the foremost cause of adult disability (Tu and Wang, 2023). In China, stroke affects 10.36 million individuals aged 40 and above, with an annual increase of 8.7%. Each year sees approximately 2.5 million new cases and 1.5 million deaths, constituting nearly 10% of total disease-related mortality (Wang and Zhang, 2015). Beyond classic symptoms like motor and speech impairments, post-stroke sleep disorders have emerged as a significant non-typical complication. These conditions manifest in diverse ways, including insomnia, daytime sleepiness, sleep apnea, and circadian rhythm disorders (Yan et al., 2024). The prevalence rates during acute, subacute, and chronic phases are 40.7%, 42.6%, and 35.9%, respectively (Hasan et al., 2021), with 56.7% occurring in ischemic stroke patients and 42.3% in hemorrhagic stroke cases (Zhang K. et al., 2024). The clinical manifestations of post-stroke sleep disorders evolve with disease progression. Studies (Gottselig et al., 2002) indicate that during the acute phase (≤10 days), patients exhibit significantly reduced power and coherence of sleep spindles (associated with N2-N3 stages), leading to impaired slow-wave sleep architecture. The subacute phase (7–90 days) is characterized by circadian rhythm disturbances, primarily caused by damage to circadian rhythm regulatory centers such as the thalamus and hypothalamus. This results in abnormal melatonin secretion, disrupting synchronization between endogenous circadian rhythms and environmental factors (Zhang et al., 2025). In the chronic phase (over 90 days), most patients present with persistent insomnia or hypersomnia (Zhang K. et al., 2024). These sleep disorders pose significant risks, impeding neurological rehabilitation and correlating with cognitive decline, increased depression risk, and stroke recurrence (Zhao et al., 2021). This complexity necessitates targeted interventions in clinical practice.

The brain-gut axis (BGA) constitutes a complex bidirectional communication network between the gut and central nervous system, where the gut microbiota regulates brain function through immune, neural, and endocrine pathways (Loh et al., 2024; Mayer et al., 2014). In the immune pathway, dysbiosis reduces short-chain fatty acids (SCFAs), impairing regulatory T cell function, triggering systemic inflammation, activating microglia, and disrupting sleep-related neural circuits (Shen and Sun, 2021; Sinagra et al., 2020). Through the neural pathway, microbial metabolites act via the vagus nerve on the brainstem solitary nucleus and suprachiasmatic nucleus (SCN) to regulate sleep-wake rhythms (Bravo et al., 2011). In the endocrine pathway, the BGA modulates serotonin and melatonin secreted by intestinal chromaffin cells, with these substances influencing cerebral sleep homeostasis through circulation (Sun et al., 2021). Building on this framework, our study integrates the microbiome-immune-neurotransmitter network to elucidate BGA-mediated mechanisms in post-stroke sleep disorders, providing theoretical foundations for targeted gut microbiota interventions to improve patient sleep quality and prognosis.

## 2 Changes in microbiome and their effects after stroke

### 2.1 Pathways of stroke effects on intestinal function

Stroke affects intestinal function through interactions between the neuroendocrine and autonomic nervous systems. These systems work in concert to disrupt gut function, with the hypothalamic-pituitary-adrenal (HPA) axis playing a pivotal role. The stress response following stroke causes a sharp rise in glucocorticoid levels (particularly cortisol), and persistent elevation damages intestinal barrier integrity. This manifests as reduced expression of tight junction proteins like occludin and claudin-5, increased apoptosis of intestinal epithelial cells, and heightened intestinal permeability–collectively termed “leaky gut” (Li et al., 2019; Yang et al., 2024). Studies (Wan et al., 2024; Wells et al., 2017; Zhang et al., 2010) indicate elevated plasma cortisol levels in stroke patients, likely triggered by HPA axis activation, which compromises intestinal barriers. The resulting increase in circulating levels of zonulin, a biomarker of tight junction disruption, confirms enhanced gut permeability, thereby demonstrating cortisol’s detrimental effect on the intestinal barrier. Moreover, animal studies (Lin et al., 2020) have further confirmed that exogenous stressors such as restraint stress can simulate the post-stroke condition by activating the HPA axis and elevating glucocorticoid levels, directly resulting in significantly delayed intestinal motility in mice. This finding underscores stress itself as a critical factor in triggering intestinal dysmotility. Dysregulation of the autonomic nervous system is another major contributor. Stroke lesions, particularly in the insula and brainstem, disrupt central autonomic control, leading to sympathetic overactivation and reduced vagal tone (Cheng et al., 2020). Research (Nagai et al., 2021) indicates that that right insular stroke enhances sympathetic tone, while brainstem injuries are strongly linked to impaired gut sensation and motility. Sympathetic excitation induces intestinal vasoconstriction, reducing blood flow and compromising nutrient supply–a process associated with sympathetic-mediated microcirculatory dysfunction and cellular injury (Prame Kumar et al., 2025). Reduced vagal tone slows intestinal peristalsis, induces gastrointestinal paralysis, and decreases acetylcholine release in the enteric nervous system, further exacerbating intestinal motility disorders (Veldman et al., 2025). Animal studies (Ameer et al., 2010) also confirm that in middle cerebral artery occlusion (MCAO) model rats, intestinal transit time was longer than the control group at 2 and 12 h post-stroke, demonstrating significant effects of stroke on intestinal motility.

### 2.2 Post-stroke intestinal flora changes

Following a stroke, the diversity of gut microbiota undergoes significant changes. Studies (Xie et al., 2023; Zhang et al., 2023) confirm that compared to healthy individuals, stroke patients exhibit markedly reduced α-diversity in their gut microbiota (measured by species richness and evenness within communities). This decline in microbial diversity weakens the stability and functionality of the intestinal ecosystem, reducing its resistance to disturbances. Consequently, patients become more vulnerable to pathogenic bacteria, potentially triggering gastrointestinal disorders and systemic inflammation. These changes may also disrupt metabolic functions, leading to abnormal production of metabolites (Xie et al., 2023). Additionally, post-stroke β-diversity in gut microbiota–referring to differences between microbial communities–may vary depending on stroke type, severity, and individual characteristics (Xie et al., 2023). Investigating these variations helps comprehensively understand microbial alterations and provides clues for identifying specific biomarkers. Post-stroke, the composition of gut microbiota shows marked shifts: beneficial bacteria like *Bifidobacterium* and *Lactobacillus* experience significant reductions in abundance, while opportunistic pathogens such as Enterobacteriaceae increase in numbers (Luo et al., 2023). Beneficial bacteria ferment carbohydrates to produce SCFAs, which lower intestinal pH levels to inhibit pathogenic bacteria, stimulate gut immunity, and synthesize vitamins to enhance nutrient absorption (Aggarwal et al., 2021; Hadji and Bouchemal, 2022). A decline in beneficial bacteria disrupts the balance of gut microbiota, weakening the intestinal barrier and immune functions (Chen et al., 2025; Zhang et al., 2023). The proliferation of opportunistic pathogens poses significant risks. Under normal conditions, these bacteria remain scarce, but when microecological balance is disrupted, they multiply rapidly and release harmful substances like endotoxins. These toxins entering the bloodstream trigger systemic inflammation and tissue damage (Xu et al., 2021), such as activating the Toll-like receptor 4 (TLR4) signaling pathway to release pro-inflammatory factors like IL-6 and TNF-α, exacerbating neuroinflammation and tissue injury (Mao et al., 2023). Additionally, alterations in microbial composition may affect the metabolism and functions of other bacterial groups. For instance, compromised symbiotic relationships disrupt normal microbial growth and metabolism, leading to overall functional disorders.

### 2.3 Stroke-a vicious circle of bacterial flora disorder

#### 2.3.1 Intestinal barrier damage aggravates bacterial imbalance

The intestinal barrier normally prevents lipopolysaccharide (LPS) from entering the bloodstream during healthy conditions. However, when disrupted by factors such as gut microbiota imbalance, aging, dietary changes, or pathogen exposure, LPS can translocate from the gut into the bloodstream (Zhang Y. et al., 2024), a condition known as “leaky gut.” This phenomenon not only results from post-stroke stress but may also exacerbate microbial dysbiosis. Impaired intestinal barriers allow bacteria and their metabolites to enter the bloodstream, triggering systemic inflammation while suppressing beneficial bacteria and creating opportunities for opportunistic pathogens to proliferate (Zhu and Xu, 2025). For instance, altered oxygen levels and pH in the gut due to barrier damage favor the growth of facultative anaerobes like Enterobacteriaceae, increasing their proportion and disrupting the microecological balance. The normally oxygen-deprived, acidic gut environment then promotes the growth of anaerobic bacteria such as *Bifidobacterium*. Subsequent oxygen influx and pH elevation further enable facultative anaerobes to thrive (Dang et al., 2021; Wang et al., 2023). Moreover, the bacteria and metabolites entering the bloodstream activate the immune system, inducing systemic inflammation that conversely damages the intestinal barrier, forming a “damage-inflammation-reinjury” vicious cycle (Di Tommaso et al., 2021). This continuous cycle worsens microbial dysbiosis and barrier damage, impairing systemic functions and exacerbating stroke patients’ clinical outcomes.

#### 2.3.2 Alteration of microbial metabolites

Post-stroke intestinal microbiota alterations lead to changes in metabolites. SCFAs, as crucial metabolic products, experience significant reductions after stroke. SCFAs not only serve as an energy source for intestinal epithelial cells but also regulate immunity and maintain the intestinal barrier function. When their levels decrease, the induction and activation of regulatory T cells are weakened, leading to immune imbalance and exacerbated inflammation (Xu et al., 2021). Bile acid metabolism may also be altered. Gut microbiota participate in bile acid conversion to generate secondary bile acids, which regulate lipid metabolism, glucose metabolism, and immune functions (Wang et al., 2022; Xie et al., 2022). Dysbiosis may disrupt bile acid metabolism, affecting systemic metabolism and immunity, thereby worsening complications. Tryptophan metabolism might also be impacted. Microbiota convert tryptophan into indole derivatives that exhibit anti-inflammatory and barrier-regulating effects (Xie et al., 2022). Dysbiosis may reduce beneficial substances while increasing harmful byproducts, exacerbating inflammation. Additionally, harmful metabolites like LPS may accumulate. As a component of Gram-negative bacterial cell walls, LPS activates the immune system upon entering the bloodstream (Dong et al., 2024), triggering systemic inflammation. Inflammatory factors induce endothelial cell apoptosis and increase oxidative stress. Moreover, LPS can directly bind to receptors on cells of the blood-brain barrier (BBB), leading to a loss of barrier function (Peng et al., 2021). This effect is primarily attributed to the specific interaction between LPS and the TLR4 and its co-receptor myeloid differentiation factor 2 (MD-2) complex on the BBB cell surface (Shimazu et al., 1999). The binding of LPS to the TLR4/MD-2 complex activates downstream signaling pathways like NF-κB, which in turn induces the downregulation or aberrant redistribution of key endothelial tight junction proteins–including claudin-5, occludin, and ZO-1–leading to an increase in paracellular permeability (Gu et al., 2018). Furthermore, LPS can induce the production of pro-inflammatory cytokines and reactive oxygen species (ROS) in endothelial cells (Shen et al., 2025), which further compromises BBB integrity and exacerbates brain tissue damage. In conclusion, abnormal metabolites are an important link in the vicious circle, which will affect the body function through a variety of ways, leading to the deterioration of the disease and poor prognosis. The regulation of metabolite levels may become the target of treatment.

## 3 Interacting mechanisms of microbiome-immune-neurotransmitter networks

### 3.1 Microbiome affects neural function through immune pathway

Under normal conditions, SCFAs such as acetic acid, propionic acid, and butyric acid are produced through the fermentation of dietary fiber by gut microbiota. These compounds promote the differentiation and proliferation of regulatory T cells (Tregs), enhancing their ability to secrete immunosuppressive factors like IL-10 and TGF-β, thereby suppressing excessive immune responses (Wang X. et al., 2024). Post-stroke dysbiosis reduces SCFAs production, inhibits Treg cell differentiation and function, leading to decreased secretion of IL-10 and TGF-β, which diminishes the body’s immune suppression capacity (Zhang Y. et al., 2024). This triggers enhanced activity of pro-inflammatory T cells (Th17 and γδT cells) that release large amounts of inflammatory factors like IL-6 and TNF-α (Luo et al., 2023). Neuroinflammation damages neurons and neural circuits, impairing nervous system function. It also breaches the BBB, allowing more harmful substances to enter the brain and exacerbate injury (Braniste et al., 2014). When SCFA levels are insufficient, immune cells (macrophages and dendritic cells, DCs) secrete increased amounts of pro-inflammatory factors like TNF-α and IL-6 while reducing anti-inflammatory factors such as IL-10, resulting in uncontrolled inflammation (Xi et al., 2025). Excessive maturation of DCs leads to T cell activation and the initiation of inflammatory responses, such as Th1/Th17 differentiation, thereby exacerbating tissue damage (Li et al., 2017). Damage to the intestinal barrier allows LPS in the gut to enter the bloodstream more easily. As a key component of Gram-negative bacterial cell walls, LPS exhibits strong immunogenicity (Dong et al., 2024). Once in the blood, LPS binds to TLR4 on immune cells like macrophages and microglia, or on brain endothelial cells, activating the NF-κB signaling pathway and triggering massive release of pro-inflammatory factors such as TNF-α and IL-6 (Chen et al., 2024). The excessive release of IL-6 and TNF-α exacerbates neuroinflammation. These factors cross the damaged BBB into the brain, inducing microglia to adopt an inflammatory phenotype. They release neurotoxic substances like nitric oxide (NO) and prostaglandin E2 (PGE2), causing oxidative damage and energy metabolism disorders in neurons (Ma et al., 2024), which disrupt neural circuits and impair sleep-related neural regulation. Research (Lyu et al., 2004) shows that NO excess directly attacks nerve cells as ROS, while insufficient NO fails to maintain cerebral vascular tone, leading to reduced blood flow and hypoxic injury. PGE2 promotes the release of other inflammatory factors like IL-1β and MMP-13, initiating inflammatory cascades (Dou, 2012). These combined effects ultimately cause neuronal damage, disrupt neural circuit integrity, and interfere with sleep-wake cycle regulation.

### 3.2 Immune-mediated neurotransmitter disorders

Immune-mediated neurotransmitter dysregulation plays a pivotal role in post-stroke sleep disorders, with inflammatory factors suppressing serotonin 5-hydroxytryptamine (5-HT) synthesis being a critical component. 5-HT, which regulates sleep and emotional states, is primarily synthesized by the raphe nucleus group in the brainstem through tryptophan hydroxylase catalysis. In inflammatory conditions, interleukin-6 (IL-6) and tumor necrosis factor-α (TNF-α) inhibit tryptophan hydroxylase activity – the rate-limiting enzyme for 5-HT synthesis. This inhibition reduces 5-HT production (Wang and Jiang, 2023). As 5-HT typically promotes wakefulness and inhibits rapid eye movement (REM) sleep, decreased levels may impair arousal function and cause abnormal REM regulation (shortened or fragmented REM periods), thereby disrupting sleep cycle stability. Post-stroke pontine injury may overactivate 5-HT ergic neurons, leading to abnormal 5-HT elevation and insomnia. This highlights conflicting effects of different brain region lesions on 5-HT (Han et al., 2024). Conversely, reduced 5-HT synthesis weakens its arousal-promoting effect, potentially causing drowsiness and impaired REM regulation. These dysregulations adversely affect both sleep quality and daytime functioning, hindering recovery. Additionally, since 5-HT is involved in emotional regulation, its deficiency may induce depression and anxiety, which in turn exacerbate sleep disorders, creating a vicious cycle (Hu et al., 2018). Clinical observations show higher prevalence of sleep disorders among post-stroke patients with comorbid depression, confirming the interconnected roles of 5-HT and other neurotransmitters.

Inflammatory reactions can impair astrocyte function, leading to excitotoxicity. In neuroinflammation, activated astrocytes show significantly reduced expression of glutamate transporters such as GLT-1/EAAT2 and GLAST/EAAT1, accompanied by morphological changes, hyperplasia, and excessive release of cytokines and inflammatory mediators that decrease glutamate uptake (Liu and Dong, 2024). Normally, astrocytes rapidly take up synaptic glutamate through GLT-1 and GLAST transporters, converting it into glutamine before returning to neurons to maintain glutamate homeostasis and prevent neuronal damage from excessive concentration (Wu et al., 2023). Extracellular glutamate accumulation overactivates NMDA and AMPA receptors, triggering calcium influx (Liu and Dong, 2024). Calcium overload activates enzymes like calmodulin, inducing neuronal apoptosis or necrosis (Yan et al., 2025), which damages neuronal structure-function integrity, disrupts signal transmission, and affects sleep-wake cycle regulation. Glutamate serves as a key activator for arousal-related neural nuclei; abnormal elevation of its concentration overstimulates these nuclei, causing sleep initiation difficulties or sleep architecture disruption (Yan et al., 2025). Additionally, impaired astrocyte function affects the metabolic regulation of other neurotransmitters like γ-aminobutyric acid (GABA), directly reducing GABA synthesis and weakening inhibitory neurotransmission (Ji et al., 2024), thereby exacerbating sleep disturbances.

### 3.3 Direct regulation of nervous system by microbiome

#### 3.3.1 Bacterial metabolites affect sleep rhythm through vagus nerve

Neuroactive metabolites derived from the gut microbiota, such as GABA and histamine, function as critical signaling molecules in gut-brain axis communication. These microbial products transmit signals via the vagus nerve to the central nervous system, where they directly participate in the fine-tuned regulation of the sleep-wake cycle. Of particular interest is the growing emphasis in recent research on dynamic alterations in the microbial sources of these metabolites–especially under stress conditions–in addition to their intrinsic neuromodulatory functions. This shift in focus enables a more profound understanding of the interplay between gut microbiota and neural processes. Empirical evidence indicates that (Wang, 2017) chronic stress induces significant restructuring of the gut microecology, characterized by a decline in the abundance of GABA-producing taxa, such as *Lactobacillus* and *Bifidobacterium*, alongside a potential proliferation of bacterial groups with histamine-producing capabilities, including *Enterococcus*. Such structural disruption within microbial communities responsible for key neuroactive molecules is likely to perturb metabolic homeostasis, thereby establishing a biological basis for stress-related sleep disturbances. This mechanistic insight underscores the fundamental role of the gut microbiota in the regulation of sleep. As a primary inhibitory neurotransmitter in the central nervous system, GABA reduces brain excitability by suppressing glutamatergic neuron activity, promoting deep sleep (Fan et al., 2021). Gut microbiota metabolites SCFAs, neurotransmitters stimulate enteroendocrine cells (EECs), activating vagal nerve terminals. Signals are then transmitted through the solitary nucleus to hypothalamic regions like the SCN, which regulates sleep (Zhao et al., 2024). The SCN serves as the biological clock control center, where GABA and other inhibitory signals may enhance sleep drive and promote sleep initiation and maintenance (Zhao et al., 2024). Histamine is associated with arousal; its abnormal overproduction may prolong wakefulness and reduce sleep, while deficiency can cause sleep interruption, increased REM sleep, and arousal deficits (Thakkar, 2011). Additionally, 5-HT acts as an arousal promoter that inhibits REM sleep and serves as a precursor to melatonin, which regulates circadian rhythms and sleep. Gut microbiota imbalance can lead to decreased 5-HT levels, causing insomnia. The microbiota also produces 5-HT precursors and dopamine precursors, which may influence central neurotransmitter balance through the vagus nerve to regulate sleep rhythms (Gui et al., 2020). Research (Luo and Jin, 2014) shows that exogenous GABA partially restores sleep rhythms in gut microbiota dysbiosis animal models, with this recovery effect disappearing after vagus nerve transection, confirming that this regulation depends on the vagus nerve pathway.

#### 3.3.2 Abnormal activation of intestinal-brain signaling pathway

Changes in gut microbiota metabolites can disrupt neurotransmitter balance, leading to abnormal activation of the gut-brain signaling pathway. Beyond the vagus nerve, these metabolites also reach the brain through circulation pathways, affecting neurotransmitter synthesis, release, and metabolism. SCFAs like acetate, propionate, and butyrate can cross the BBB to enhance GABA synthesis and release, thereby improving sleep quality. Reduced SCFAs levels impair GABA production, causing excessive brain activity and sleep disturbances (Yang et al., 2025; Zhou et al., 2024). In stroke patients with post-stroke depression, gut microbiota imbalance reduces SCFAs while decreasing serum serotonin (5-HT) levels, exacerbating sleep disorders (Sun et al., 2024). Certain gut metabolites regulate dopamine, norepinephrine, and arousal-related neurotransmitters. Imbalanced levels of these neurotransmitters may trigger hyperarousal or hypersomnia (Cai et al., 2023). Abnormal activation of the gut-brain pathway also affects the HPA axis. Metabolites like SCFAs and LPS stimulate the hypothalamus to release corticotropin-releasing hormone (CRH), activating the HPA axis and increasing cortisol secretion. Elevated cortisol disrupts circadian rhythms and suppresses deep sleep (Yin, 2022). The abnormal activation of the gut-brain signaling pathway will break the balance of neurotransmitters, and sleep disorders will inhibit the repair and regeneration of nerve cells, which will further damage the intestinal motility function and the balance of the flora. The imbalance of the flora will aggravate the abnormal gut-brain signaling, leading to the continuous deterioration of the sleep structure (Feng et al., 2021).

## 4 Post-stroke sleep disorders and neurotransmitter imbalance

### 4.1 Neuroendocrine and autonomic nervous regulation abnormalities

The stress response during the acute stroke phase overactivates the HPA axis, triggering the hypothalamus to secrete corticotropin-releasing hormone (CRH) and the pituitary gland to release adrenocorticotropic hormone (ACTH), ultimately leading to excessive cortisol secretion by the adrenal cortex (Li et al., 2024). This elevated cortisol concentration damages the intestinal barrier, causing gut leakage. It also directly affects the nervous system by regulating neurotransmitter metabolism, inhibiting serotonin (5-HT) and dopamine synthesis, which impacts neuronal excitability and plasticity, resulting in sleep structure disorders. In terms of sleep regulation, abnormally high cortisol levels disrupt circadian rhythms and suppress melatonin secretion. Melatonin regulates the sleep-wake cycle–increasing at night to promote sleep and decreasing during daytime to maintain wakefulness (Wang J. et al., 2024), leading to sleep difficulties. Clinical studies show that plasma cortisol levels in stroke patients correlate positively with sleep quality indices, while nighttime melatonin levels show negative correlation with cortisol (Sui et al., 2020). Regulating HPA axis function and reducing cortisol levels may restore melatonin rhythm and improve sleep. For instance, glucocorticoid receptor antagonists can block cortisol effects and increase melatonin secretion (Sui et al., 2020).

Stroke lesions can damage the autonomic nervous system, particularly the vagus nerve, leading to functional disorders. As a major component of the parasympathetic nervous system, the vagus nerve directly connects the gut and central nervous system. It serves as a critical communication highway, transmitting gut microbiota-derived metabolites, such as SCFAs and GABA, to the brain while simultaneously relaying central signals to the gut, regulating intestinal peristalsis, secretion, and immune responses. Post-stroke vagus nerve dysfunction disrupts this bidirectional signaling pathway (Fang and Wang, 2025). Reduced vagal tone decreases acetylcholine release, inhibiting gut motility and secretion, prolonging intestinal transit time, and altering gut microenvironment. Furthermore, vagus nerve-transmitted gut-derived signals, such as SCFAs and GABA, are crucial for maintaining the normal rhythmicity of the SCN, vagus nerve dysfunction prevents these signals from effectively modulating SCN activity, resulting in disrupted sleep-wake cycles (Bravo et al., 2011). Research shows that (Li et al., 2023) electrical stimulation of the vagus nerve reduces anxiety expression by regulating norepinephrine levels, potentially improving sleep quality. This suggests vagus nerve’s role in sleep disorders, indicating that restoring vagus nerve function could be a promising therapeutic approach.

### 4.2 Disruption of sleep-wake cycle regulation

#### 4.2.1 Hypothalamic suprachiasmatic nucleus rhythm disturbance

The SCN, serving as the core regulatory center for sleep-wake cycles, maintains a 24-h circadian rhythm through its rhythmic activity influenced by multiple factors including light exposure, neurotransmitters, and gut microbiota metabolites. By integrating internal and external signals, the SCN regulates physiological processes such as sleep, body temperature, and hormone secretion (Lu et al., 2024). Post-stroke BGA disorders disrupt SCN function through various pathways: impaired vagus nerve activity impedes signaling of gut metabolites like GABA and histamine to the SCN, disrupting rhythm regulation (Fang and Wang, 2025); increased BBB permeability allows inflammatory factors to infiltrate the CNS, activating microglia and triggering neuroinflammation that damages SCN neurons, impairing their structure and function, leading to weakened or disordered rhythms (Lyu et al., 2024); abnormal cortisol levels interfere with SCN rhythm through HPA axis hyperactivation, causing cortisol elevation that directly affects rhythm gene expression and disrupts normal circadian patterns (Fang and Wang, 2025); melatonin suppresses the firing activity of SCN neurons, for instance by reducing neuronal excitability, thereby contributing to the stabilization of circadian rhythm phase and amplitude. Reduced melatonin secretion consequently diminishes SCN rhythm stability (Hardeland, 2012a). When the SCN fails to regulate circadian rhythms properly, it disrupts the sleep-wake cycle regulation system. This manifests as circadian rhythm disorders, irregular sleep-wake patterns (such as daytime drowsiness and nighttime insomnia), or reversed circadian timing (Hardeland, 2012b). Such disruptions not only impair sleep quality but also interfere with immune function, metabolic processes, and other physiological systems, thereby hindering post-stroke rehabilitation. Research (Yu et al., 2024) demonstrates that light therapy regulating SCN rhythms can improve sleep disturbances in stroke patients, suggesting that restoring normal SCN rhythm function represents a therapeutic approach. Additionally, modulating gut microbiota and enhancing BGA functionality may also contribute to restoring SCN rhythm and improving sleep quality.

#### 4.2.2 Reduced REM sleep and disruption of slow-wave sleep structure

Post-stroke sleep disorders manifest as reduced REM sleep and disrupted slow-wave sleep architecture. REM sleep is closely associated with memory consolidation and emotional regulation, and its deficiency may impair cognitive recovery. Slow-wave sleep plays a crucial role in physical rehabilitation, while structural abnormalities can lead to decreased sleep quality, causing patients to experience fatigue and daytime drowsiness (Wu and Liu, 2024). Neurotransmitter imbalances (particularly reduced serotonin levels) inhibit REM sleep. Normally, serotonin remains low during REM stages, but post-stroke reductions in 5-HT synthesis, potentially in conjunction with alterations in other neurotransmitters such as dysregulated dopamine, may collectively contribute to abnormalities in REM sleep (Zhu et al., 2018). Neuroinflammation may damage REM sleep-related neural circuits. For instance, inflammation-induced injury to the pontine REM sleep regulatory center affects its generation and maintenance. Inflammatory factors disrupt neurotransmitter release pathways, interfering with normal REM sleep cycles (Li et al., 2015). Slow-wave sleep production correlates with ventrolateral preoptic nucleus (VLPO) and thalamic activity. Damage to VLPO cells (due to aging or disease) reduces both non-REM and REM sleep duration, as VLPO contains inhibitory neurotransmitters that suppress arousal systems during sleep onset (Sherin et al., 1996). BGA disorders may disrupt the function of these brain regions, potentially damaging their structure. For instance, reduced SCFAs can weaken neuroprotective mechanisms, potentially impairing neurons in the ventral raphe nucleus (VLPO) and affecting the generation of slow-wave sleep (Lu et al., 2000). Research (Xie et al., 2013) reveals a correlation between post-stroke reduction of slow-wave sleep and poor neurological recovery. During slow-wave sleep, the brain clears metabolic waste such as β-amyloid protein, promotes neural plasticity, and facilitates functional repair. Structural damage to this process hinders rehabilitation, creating a vicious cycle. By improving gut microbiota to increase SCFAs production and reduce neuroinflammation, REM sleep and slow-wave sleep structures may be restored, enhancing sleep quality and accelerating recovery. Probiotic interventions, for example, have been shown to increase REM and slow-wave sleep duration in post-stroke animals while improving sleep architecture, providing experimental evidence for clinical treatment.

## 5 Future research directions

### 5.1 Precision intervention targeting intestinal flora

Given the critical role of gut microbiota in post-stroke sleep disorders, targeted precision interventions represent a crucial direction for future research. This includes optimizing and applying therapeutic approaches such as probiotics, prebiotics, synbiotics, and fecal microbiota transplantation (FMT). Emerging evidence provides preliminary support for the efficacy of microbiota-targeted interventions. Studies have demonstrated that (Ren et al., 2024) probiotic supplementation with *Lactobacillus* and *Bifidobacterium* strains can restore stress-induced dysfunction of the HPA axis and ameliorate anxiety-like behaviors. Furthermore, specific probiotic strains, including *Lacticaseibacillus rhamnosus* JB-1, *Lacticaseibacillus paracasei* PS23, and *Ligilactobacillus gasseri* CP2305, have shown promising effects in alleviating anxiety- and depression-like phenotypes while improving sleep quality in experimental models (Liu et al., 2024), suggesting their potential applicability in mitigating post-stroke stress and associated sleep disturbances. Similarly, prebiotics such as fructooligosaccharides and inulin, as well as synbiotics, can selectively promote the proliferation of beneficial microbial populations and improve gut microbiota homeostasis, thereby amplifying the aforementioned beneficial effects (Markowiak and Śliżewska, 2017). Notably, FMT from healthy donors has been shown to partially reset dysbiotic gut environments in disease models, with concomitant improvements in anxiety-related behaviors (Zhang et al., 2022). These collective findings offer a compelling theoretical foundation and future perspective for developing microbiota-based interventions aimed at preventing and treating post-stroke sleep disorders. Future efforts should focus on developing standardized microbiota intervention protocols, conducting multi-omics studies integrating metagenomics, metabolomics, and proteomics technologies to deeply analyze molecular interactions between specific strains and host. This will enable personalized intervention strategies tailored to patients’ microbiota characteristics, genetic backgrounds, and clinical phenotypes, providing new therapeutic targets and approaches for post-stroke sleep disorder management.

### 5.2 Mechanism study of microbiome-immune-neurotransmitter network

A comprehensive investigation into the network’s interaction mechanisms is crucial for understanding the development of post-stroke sleep disorders and providing theoretical foundations for developing new therapeutic targets. Future research should elucidate the complex interactions among these three aspects at molecular, cellular, and whole-body levels. At the molecular level, research should focus on the specific binding mechanisms between microbial metabolites–such as SCFAs and tryptophan derivatives–and neurotransmitter receptors, including GABA and 5-HT receptors, as well as their regulatory effects on neuronal excitability. At the cellular level, it is essential to clarify the causal relationship between microglia polarization states (M1/M2 types) and dynamic changes in gut microbiota, along with astrocytes’ bridging role in metabolite transport and neurotransmitter circulation. Whole-body level studies should integrate multi-omics data to construct dynamic network models integrating microbiome, immune system, and neurotransmitters, thereby analyzing the temporal patterns of this network’s changes after stroke. Notably, post-stroke BBB integrity disruption may alter intracerebral microenvironment, leading to neuronal dysfunction or cell death (Dong et al., 2024). Future research could establish *in vitro* blood-brain barrier models to systematically evaluate how different microbial metabolites affect barrier permeability. Simultaneously, optogenetic and chemogenetic technologies can be employed to precisely modulate specific neural circuits, validating their regulatory effects on sleep-wake cycles and laying a solid theoretical foundation for developing targeted intervention strategies.

### 5.3 Clinical translational research

The ultimate goal of translating basic research into clinical applications is to improve sleep quality and prognosis in stroke patients. Future efforts should focus on strengthening translational research to develop BGA-based diagnostic and therapeutic strategies. By analyzing patient microbiota composition, metabolic products, blood and cerebrospinal fluid inflammatory factors, and neurotransmitters, we can identify specific biomarkers with high sensitivity and specificity to build diagnostic models for early prediction and diagnosis. For instance, detecting specific microbial ratios along with IL-6, TNF-α, and SCFAs levels could enhance diagnostic accuracy through combined predictive models. On the therapeutic front, large-scale multicenter randomized controlled trials should evaluate the efficacy and safety of targeted microbial interventions (e.g., probiotics, prebiotics, fecal microbiota transplantation). Exploring combined treatment strategies–such as integrating microbial interventions with traditional sleep disorders therapies (CBT, pharmacotherapy)–could improve outcomes. For insomnia patients, combining probiotic supplementation with sleep restriction therapy may synergistically enhance sleep quality. Similarly, depressive sleep disorder patients could benefit from integrated microbial regulation and cognitive behavioral therapy interventions. Additionally, establishing personalized treatment decision-making systems based on patients’ microbiota profiles, immune status, and clinical manifestations will enable precise treatment planning and continuous improvement of therapeutic outcomes.

## 6 Conclusion

Post-stroke sleep disorders, a prevalent and clinically significant complication, are closely associated with BGA dysregulation. This condition disrupts intestinal function through neuroendocrine and autonomic pathways, leading to microbial imbalance (reduced diversity and pathogen overgrowth), compromised gut barrier integrity, and systemic inflammation. Within this pathological cascade, the microbiome-immune-neurotransmitter network interferes with sleep regulation. Reduced SCFAs and circulating LPS trigger neuroinflammation, while inhibition of serotonin (5-HT) synthesis results in glutamate-induced excitotoxicity. Overactive HPA axis and vagus nerve dysfunction further disrupt circadian rhythm regulation, ultimately causing reduced REM sleep and disrupted slow-wave sleep architecture. Although the exact mechanisms remain debated, BGA has emerged as a critical therapeutic target. Future research should focus on deciphering the interactive mechanisms within the microbiome-immune-neurotransmitter network, developing targeted interventions such as probiotics and FMT, and validating clinical translation studies to provide innovative approaches for improving sleep quality and neurological recovery, thereby offering new hope for patients.
